# Supplementary material for: Kinetic Characteristics of Curcumin and Germacrone in Rat and Human Liver Microsomes: Involvement of CYP Enzymes
Source: Molecules. 2022 Jul 14;27(14):4482. doi: 10.3390/molecules27144482 (PMC9317718; doi:10.3390/molecules27144482)
Supplement: Supplementary file 1 [file molecules-27-04482-s001.zip › molecules-1782645-supplementary.pdf]

Table S1. The matrix-matched calibration and solvent calibration (0.01–120 μM) (mean ± SD, *n* = 3).

Compd	Matrix	Regressive equation	Correlation coefficient (r^2)	LOD (μM)	LOQ (μM)
Curcumin	solvent	y = 5603246 x + 12385	0.9992		
	RLMs	y = 5324486 x + 23266	0.9936	0.018	0.15
	HLMs	y = 5412463 x + 26482	0.9958	0.025	0.13
Germacrone	solvent	y = 2846581 x + 28365	0.9998		
	RLMs	y = 2634468 x+ 26383	0.9988	0.032	0.09
	HLMs	y = 2693386 x + 24368	0.9962	0.026	0.12

Table S2. Accuracy and precision of curcumin and germacrone in RLMs and HLMs (mean ± SD, *n* = 3).

Compd	Concn (μM)	Intra-day			Inter-day		
		Concn (μM)	Accuracy (%)	RSD (%)	Concn (μM)	Accuracy (%)	RSD (%)
Curcumin	3 (R)	2.932 ± 0.12	96.87	1.22	2.940 ± 0.38	92.31	2.98
	3 (H)	2.896 ± 0.36	94.63	1.08	2.961 ± 0.43	93.62	2.34
	10 (R)	9.561± 0.38	93.56	1.92	9.534 ± 0.48	89.67	2.83
	10 (H)	9.743 ± 0.12	93.28	2.13	9.463 ± 0.38	91.23	2.62
	80 (R)	79.36 ± 0.68	98.26	3.04	79.29 ± 057	94.53	2.12
	80 (H)	79.42 ± 0.23	96.47	2.52	79.53 ± 0.23	93.25	2.36
Germacrone	3 (R)	2.945 ± 0.72	91.32	2.34	2.864 ± 0.77	89.43	1.96
	3 (H)	2.936 ± 0.82	93.38	2.19	2.832 ± 0.98	90.68	1.89
	10 (R)	9.123 ± 0.24	89.64	1.31	9.425 ± 0.72	91.20	2.32
	10 (H)	9.423 ± 0.36	90.26	1.56	9.332 ± 0.64	92.32	1.97
	80 (R)	78.63 ± 0.32	85.53	1.84	79.12 ± 0.64	88.47	1.71
	80 (H)	79.32 ± 0.13	88.42	2.03	79.43 ± 0.12	95.43	2.32

Note: R meant RLMs and H meant HLMs

Table S3. The recovery of curcumin and germacrone (mean ± SD, *n* = 3).

Martic	Concn (μM)	Curcumin		Germacrone	
		Accuracy (%)	RSD (%)	Accuracy (%)	RSD (%)
RLMs	3.00	95.36 ± 3.65	3.42	98.96 ± 3.87	2.65
	10.00	97.36 ± 2.32	2.37	99.96 ± 1.23	1.56
	80.00	95.67 ± 4.62	2.12	98.63 ± 3.26	2.67
HLMs	3.00	98.12 ± 3.24	2.72	102.32 ± 1.84	3.42
	10.00	97.12 ± 2.86	1.92	99.23 ± 1.12	2.34
	80.00	100.46 ± 2.68	2.02	98.68 ± 1.68	2.63
